# Aerobic H_2_ production related to formate metabolism in white-rot fungi

**DOI:** 10.3389/ffunb.2023.1201889

**Published:** 2023-06-27

**Authors:** Toshio Mori, Saaya Takahashi, Ayumi Soga, Misa Arimoto, Rintaro Kishikawa, Yuhei Yama, Hideo Dohra, Hirokazu Kawagishi, Hirofumi Hirai

**Affiliations:** ^1^ Faculty of Agriculture, Shizuoka University, Shizuoka, Japan; ^2^ Research Institute for Mushroom Science, Shizuoka University, Shizuoka, Japan; ^3^ Faculty of Science, Shizuoka University, Shizuoka, Japan; ^4^ Research Institute of Green Science and Technology, Shizuoka University, Shizuoka, Japan; ^5^ Graduate School of Science and Technology, Shizuoka University, Shizuoka, Japan

**Keywords:** aerobic H_2_ production, formate dehydrogenase, oxalate metabolism, *Trametes versicolor*, white-rot fungi

## Abstract

Biohydrogen is mainly produced by anaerobic bacteria, anaerobic fungi, and algae under anaerobic conditions. In higher eukaryotes, it is thought that molecular hydrogen (H_2_) functions as a signaling molecule for physiological processes such as stress responses. Here, it is demonstrated that white-rot fungi produce H_2_ during wood decay. The white-rot fungus *Trametes versicolor* produces H_2_ from wood under aerobic conditions, and H_2_ production is completely suppressed under hypoxic conditions. Additionally, oxalate and formate supplementation of the wood culture increased the level of H_2_ evolution. RNA-seq analyses revealed that *T. versicolor* oxalate production from the TCA/glyoxylate cycle was down-regulated, and conversely, genes encoding oxalate and formate metabolism enzymes were up-regulated. Although the involvement in H_2_ production of a gene annotated as an iron hydrogenase was uncertain, the results of organic acid supplementation, gene expression, and self-recombination experiments strongly suggest that formate metabolism plays a role in the mechanism of H_2_ production by this fungus. It is expected that this novel finding of aerobic H_2_ production from wood biomass by a white-rot fungus will open new fields in biohydrogen research.

## 1 Introduction

Hydrogen gas is considered a potential sustainable energy carrier due to its advantages of high energy density, zero emissions when burning, and ease of production from various renewable sources. H_2_ can be produced from biomass materials *via* both thermochemical and biological processes. Photo- and dark fermentation are biological conversion processes by which organic substrates and/or biomass materials can be used to produce H_2_ by a diverse group of microorganisms. In dark fermentation, carbohydrates in the biomass are broken anaerobically to H_2_, CO_2_, and organic acids by hydrogen-producing anaerobes (Ghimire et al., 2015). Some anaerobic bacteria, such as those of the genera *Escherichia* and *Clostridium*, can produce H_2_ from organic acids (Mnatsakanyan et al., 2004; Matsumoto and Nishimura, 2007). Photo-fermentation of organic substrates is performed by photosynthetic bacteria. These bacteria utilize small organic acids to produce H_2_ under anaerobic conditions in the presence of light (Azwar et al., 2014). Various anaerobic eukaryotes are also able to produce H_2_ in hydrogenosomes. The anaerobic fungi *Neocallimastix* and *Piromyces* spp. are well-known H_2_ producers (Hackstein et al., 1999). These enteric fungi hydrolyze carbohydrates, and the resulting sugars are metabolized to pyruvate *via* glycolysis or to malate *via* the tricarboxylic acid (TCA) cycle (Hess et al., 2020). Hydrogenosomes metabolize pyruvate and malate to acetate for ATP generation, and H_2_ and CO_2_ are also generated by the combined ATP generation reaction (Marvin-Sikkema et al., 1993; Hackstein et al., 1999). Organic acids often function as important factors in the H_2_ production process in anaerobes.

Although hydrogenase-like (or NARF; nuclear prelamin A recognition factor, NAR1; cytosolic Fe-S cluster assembly factor) genes are widely distributed in the genomes of higher eukaryotes, their function and role remain unknown (Horner et al., 2002). It is generally thought that animal cells are unable to generate H_2_, but it is also known that molecular hydrogen has diverse biological effects in animals, including anti-oxidative stress, anti-inflammatory, and anti-allergic effects (Ohta, 2012). Higher plants are also affected by H_2_, as it improves tolerance to various abiotic stresses, including oxidative, salt, and desiccation stress (Li et al., 2018). Additionally, it is thought that H_2_ improves physiological processes such as growth and development in higher plants and interacts other signaling molecules. Some early studies demonstrated hydrogenase-mediated H_2_ production in seedlings of some higher plants under sterile conditions (Renwick et al., 1964; Torres et al., 1986). Some recent reports indicated that plant hormones and abiotic stresses promote endogenous H_2_ release in higher plants and that H_2_ signaling induces plant antioxidant defenses and enhances salt tolerance (Xie et al., 2012; Zeng et al., 2013). Although details of the H_2_ production and H_2_ signaling pathways remain unclear, molecular H_2_ seems to play a very important role in stress responses in higher eukaryotes.

White-rot fungi are unique microorganisms that are capable of degrading all main wood components, cellulose, hemicellulose, and lignin. During lignin degradation, white-rot fungi produce various reactive oxygen species (ROS) and radicals, such as radical mediators (Dashtban et al., 2010). White-rot fungi also produce oxalate to release and mineralize excessive carbon in order to grow on woody materials that have an extremely high C/N ratio (Shimada et al., 1994). Based on these data, the authors predicted that white-rot fungi, which are always exposed to oxidative stress during wood decay and have excellent ability to metabolize organic acids, can produce H_2_ in conjunction with organic acid metabolism to enhance oxidative stress tolerance. In this study, it was evaluated the H_2_ production ability of various white-rot fungi during wood decay and investigated the underlying production mechanism.

## 2 Materials and methods

### 2.1 Fungal strains

White-rot fungi, *Pleurotus ostreatus* NBRC 33211, NBRC 104981, *Trametes hirsuta* NBRC 104984, NBRC 106840, and *Trametes versicolor* NBRC 104985, NBRC 106839 were obtained from the National Institute of Technology and Evaluation, Japan. *Phanerochaete chrysosporium* ME-446 (ATCC 34541) and *Phanerochaete sordida* YK-624 (ATCC 90872) were obtained from the American Type Culture Collection, USA. *Ceriporia lacerata* K-70 (accession number [AN] of internal transcribed sequence [ITS]: LC312413), *Phanerochaete* sp. K-64 (AN-ITS: LC710144), K-91 (AN-ITS: LC710143), K-97-2 (AN-ITS: LC710142), M-4 (AN-ITS: LC710145), *Schizophyllum commune* M-21 (AN-ITS: LC710146), *T. hirsuta* M-9 (AN-ITS: LC710150), *T. versicolor* K-39 (AN-ITS: LC710147), K-41 (AN-ITS: LC312415), K-86 (AN-ITS: LC710148), M-24 (AN-ITS: LC710149) and unidentified K-89 were isolated from naturally decaying wood samples and identified based on their ITS, following a previous report (Mori et al., 2018).

### 2.2 Test of H_2_ evolution from wood meal

All fungal strains were grown on PDA at 30°C. Two mycelial discs (10 mm diameter) were punched from the edge of the mycelia and placed into a 70-mL serum vial containing 0.5 g of extractive-free beech or cedar wood meal (80-100 mesh, moisture content: 80%). After 5 days of pre-incubation at 30°C under atmospheric pressure, the inoculated vial was sealed with a butyl rubber plug to limit the O_2_ supply and prevent H_2_ diffusion. The sealed vial was incubated for 14 days at 30°C, and then the headspace gas was sampled, and H_2_ production was analyzed by gas chromatography on an instrument equipped with a thermal conductivity detector (GC-TCD), as previously reported (Mori et al., 2016).

### 2.3 Characterization of H_2_ production activity of *T versicolor* K-41

To elucidate the relationship between O_2_ and H_2_ production by *T. versicolor* K-41, the experiments described below were performed. First, 5-day pre-cultures of *T. versicolor* K-41 on 0.5 g of cedar wood meal (80-100 mesh, moisture content: 80%) in serum vials were incubated stationary at 30°C after sealing with a butyl rubber septum, and the flask headspace gas was analyzed by GC-TCD every 3 days. After 15 days of incubation, the headspace gas was flushed with 10 mL of pure O_2_ or N_2_, and then incubation and headspace analysis were continued. To clarify the effect of oxygen concentration on H_2_ production by *T. versicolor* K-41, H_2_ production experiments under different O_2_ concentration conditions were performed. Sealed cedar wood cultures of *T. versicolor* K-41 were prepared as described above. The O_2_ concentrations in vials headspace were adjusted to approximately 2.5%, 6.3%, 12.5%, 25% and 50% by O_2_ flush, following replacing the headspace gas with pure N_2_ gas. After 14 days incubation at 30°C, the headspace gas was analyzed.

Two types of cedar wood meal cultures were prepared (0.5 g of cedar wood meal in a 70-mL serum vial). For the first cultures, 0-1.0% (g/g) CaCO_3_ was mixed thoroughly with cedar wood meal, and then water was added to adjust the moisture content to 80% before autoclaving. Two PDA discs of *T. versicolor* K-41 were inoculated on the cedar wood meal and then pre-incubated for 5 days at 30°C. After an additional 9 days of incubation with sealing, the headspace gas was analyzed. For the second culture, two PDA discs of *T. versicolor* K-41 were inoculated on wood meal medium without CaCO_3_ and pre-incubated for 5 days at 30°C. Then, 200 µL of 15 mM (or 0-120 mM) organic acid salt solution (pH 4.5, sodium acetate, formate or oxalate) or water was added to 5 places (40 µL each) in the wood cultures. The vials were then sealed and incubated for an additional 14 days at 30°C, followed by headspace gas analysis.

Two PDA discs of *T. versicolor* K-41 were inoculated in 5 mL of T-medium (2 g/L glucose, 1 g/L yeast extract, 1 g/L KH_2_PO_4_, 0.2 g/L [NH_4_]_2_SO_4_, and 0.5 g/L MgSO_4_·7H_2_O [pH 4.5]) with or without 0.1% CaCO_3_ in 70-mL serum vials and pre-incubated for 5 days. After 9 days of incubation with sealing, the headspace gas was analyzed in the same manner described above.

### 2.4 RNA-seq analysis

Cedar wood medium was used for H_2_ production, and T-medium was used as a non–H_2_-producing medium. *Trametes versicolor* K-41 was incubated aerobically for 10 days on cedar wood medium, and total RNA was extracted from 200 mg (wet) of culture by bead beating in 0.7 mL of Plant RNA Purification Reagent (Invitrogen). The RNA was then purified using an RNeasy Plant Mini kit (Qiagen) plus an RNase-free DNase set (Qiagen) following the manufacturer’s protocol. Total RNA was cleaned and concentrated using NucleoSpin RNA Clean-up XS (TaKaRa Bio Inc.) following the manufacturer’s protocol. Under non–H_2_-producing conditions, 10 mycelial discs were inoculated on 50 mL of T-medium and incubated for 7 days, and total RNA was extracted as described above. RNA quality was assessed by agarose gel electrophoresis and determination of the OD_260_/OD_280_ ratio.

For RNA-seq analysis, library construction and sequencing were entrusted to Macrogen-Japan Co. The libraries were constructed using a TruSeq Stranded mRNA Library prep kit (Illumina Inc.) according to the manufacturer’s protocol. Transcriptome sequencing of paired-end reads (150 bp) was performed using a NovaSeq 6000 system (Illumina). The raw reads (DRR374972-75) were cleaned using Trimomatic v. 0.38 to remove adapter sequences and low-quality bases (quality scores <30) and reads shorter than 100 nt (Bolger et al., 2014). Resultant high-quality paired-end reads were aligned to the *T. versicolor* FP-101664 SS1 genome sequence (GCF_000271585.1) using HISAT2 v. 2.1.0 (Kim et al., 2015). Transcript abundance was estimated using FeatureCounts v. 2.0.0 (Liao et al., 2014). Differentially expressed genes (DEGs) were identified using the likelihood-ratio test implemented in the edgeR package v. 3.16.4 (Robinson et al., 2010). DEGs were defined by a log2 fold-change (logFC) >1 and logFC <−1 with a false discovery rate (FDR) <0.05. To identify significantly over- and under-represented biological features associated with H_2_ production, gene ontology (GO) enrichment analysis was performed by parametric analysis of gene set enrichment (Kim and Volsky, 2005) based on the logFC between the H_2_-producing and non–H_2_-producing conditions.

### 2.5 Effects of O_2_ level and CaCO_3_ on expression of *Tvhyd* and *Tvfdh*

*Trametes versicolor* K-41 was pre-incubated for 5-day on cedar wood meal culture with or without 0.5% CaCO_3_, as described above. After sealing all cultures following removal of PDA pellets, the cultures (without CaCO_3_) were divided into three groups: O_2_ purge (O_2_ concentration fitted to average 80%), N_2_ purge (O_2_ concentration <0.5%), and control (without gas purge). After 7 days of incubation, the headspace gas was analyzed, and total RNA was extracted from 200 mg (wet) of culture. Total RNA (100 ng) was employed for cDNA synthesis using PrimeScript II reverse transcriptase (TaKaRa Bio). Quantitative PCR was performed on a Lightcycler 96 (Roche) system using TB Green Premix Ex Taq II (Tli RNaseH Plus, TaKaRa Bio Inc.). Gene-specific primers for the hydrogenase-like gene (AN: LC710151, *Tvhyd*; 5’-cgcaaatagcacatcgaccg-3’/5’-gacgtgatacacccactgca-3’), formate dehydrogenase (AN: LC710153, *Tvfdh*; 5’-tactccgccggaatgaagattgt-3’/5’-aactcatggccctgctcctc-3’), and glyceraldehyde-3-phosphate dehydrogenase (AN: LC710152, *Tvgpd*; 5’-cgctgtgaacgaccccttca-3’/5’-cttgccgtccttgacctcga-3’) were designed. Expression levels were calculated according to the ΔΔCq method using *Tvgpd* as the reference gene. Relative H_2_ production and expression were calculated by comparison to values of the control.

### 2.6 Tvfdh expression in *Escherichia coli*

*Tvfdh* cDNA was amplified to attached *Kpn*I and *Bam*HI sites by PCR using primers (5’-actggtaccatgctcgccggcatctcgtc-3’ and 5’-ataggatcctcacttgcgctggccgtacg-3’), then amplified PCR product was ligated between the corresponding restriction sites of pCold I vector (TaKaRa Bio). Constructed vector was transformed into Chaperone Competent Cells pGro7/BL21 (TaKaRa Bio) following manufacture’s protocol. The transformed *E. coli* was incubated in 3 mL of LB medium containing 0.5 mg/mL arabinose, 20 µg/mL chloramphenicol and 50 µg/mL ampicillin, at 37 °C, 200 rpm. After OD_600_ was reached at 0.4, the culture was cooled to 4 °C, then 1.0 mM ATP and 0.1 mM isopropyl-β-D-thiogalactopyranoside (IPTG) were added. The culture was incubated for 15 h, at 15 °C, and bacterial cells were recovered by centrifugation (10,000 × *g*, 10 min, 4 °C). Recovered cells were disrupted by bead beating (Micro smash MS-100, Tomy Seiko Co., LTD.), and cell-free extract recovered by addition of 0.1 M NaCl containing 0.1 M Tris-HCl (pH 4.5) was used for FDH activity test. FDH activity was determined by increase in absorbance at 340 nm due to formation of nicotinamide adenine dinucleotide (NADH) in the reaction mixture (0.75 mL) contained 75 mM potassium phosphate (pH 6.5), 160 µM β-NAD^+^, 20 mM sodium formate and 100 µL cell-free extract (Watanabe et al., 2005).

### 2.7 Construction of pHyg^r^ and pTvfdh

Restriction sites were attached to *Tvgpd* terminator region by 2 step of PCR reactions, using primers (5’-ggcgcgccagatctgttagcacggagta-3’/5’- gtagattctgtttggtgtgcac-3’) for first PCR and (5’-tctagaggatccggcgcgccagatct-3’/5’- gtagattctgtttggtgtgcac-3’) for second PCR. The PCR product was TA-cloned into pMD20 vector (TaKaRa Bio), and the resulting plasmid was digested at *Nde*I site. Tagged sequence for In-fusion reaction was attached to *Tvgpd* promoter region by PCR using following primers; 5’-gatctactagtcatactgagatgacctccatagc-3’ and 5’-ctctagaaatccatagtggatgtggtggatgg-3’. The tagged *Tvgpd* promoter was direct cloned into the NedI digested plasmid by In-fusion reaction using In-fusion HD cloning kit (TaKaRa Bio) following manufacture’s protocol. The constructed plasmid that having *Asc*I and *Bgl*II sites between *Tvgpd* promoter and terminator regions was designated p*Tvgpd*-pro/ter.

Hygromycin resistant gene (*hyg* ^r^) sequence then was redesigned based on *P. chrysosporium* high-frequency codon usage, and the resulting optimized gene was synthesized by GeneScript Japan, Inc. The *Asc*I and *Bgl*II restriction sites were attached to *hyg* ^r^ using primers; ggcgcgccatgaagaagcccgagc and agatctttactccttggcgcgt. p*Tvgpd*-pro/ter was digested by *Asc*I and *Bgl*II. Then the PCR product was ligated into corresponding restriction enzyme sites of p*Tvgpd*-pro/ter (pHyg^r^). Tvfdh gene was PCR amplified (primers: 5’-agaggatccggcgcgatgctcgccggcatctcgtc-3’/5’-aacagatctggcgcgtcacttgcgctggccgtacg-3’). The resulting product was used for In-fusion reaction to clone into *Asc*I digested pT*vgpd*-pro/ter to construct plasmid *Tvfdh* is under control of *Tvgpd* promoter (pTvfdh hereafter).

### 2.8 Homologous recombination of *Tvfdh*

*T. versicolor* K-41 protoplasts were prepared following method in previous report with some modification. Briefly, *T. versicolor* K-41 was precultured in liquid CYM medium at 30 °C for 7 days. The culture including mycelium was homogenized and 25 ml homogenate was added to 25 mL fresh CYM medium in 500-ml Erlenmeyer flask. The culture was incubated at 30 °C for 3 days, then mycelium was recovered by filtration. Mycelium was resuspended in 0.5M MgOsm (0.5 M MgSO_4_·7H_2_O in 10 mM 2-morpholinoethanesulfonic acid (MES), pH 6.1), following addition of 2 volume of 0.5M MgOsm containing with 5% lysing enzyme (Sigma-Aldrich) and cellulase Onozuka RS (Yakult Pharmaceutical Ind. Co., LTD.). The mixture was incubated at 30 °C, 150 rpm for 16 h. After the reaction, MgSO_4_ concentration in the solution was adjusted 1.0 M by gently addition of 2.0 M MgOsm. The crude protoplasts solution was layered onto 10 mM MES contained 1.0M sorbitol (SorbOsm), and centrifuged (1,450 × *g*, 15 min, 4 °C) to removed undigested mycelial debris. Protoplasts accumulated on interface were recovered and washed 2 times with SorbOsm. Finally, protoplasts were resuspended in SorbOsm to fit 0.5 to 0.8 × 10^8^ cells/mL.

Protoplasts were co-transformed with pHyg^r^ and pTvfdh. Plasmids (10 µg each) and 40 mM CaCl_2_ in 300 µL SorbOsm was combined with 500 µL of protoplasts solution. The mixture was incubated for 30 min at 4 °C, and then gently mixed with equal volume of PEG solution (40% PEG #4000, 10 mM CaCl_2_ in Tris-HCl, pH 7.0). After additional 30 min incubation at 4 °C, the diluted transformation solution with 5mL SorbOsm was mixed with 75 mL of regeneration medium (0.75 M sucrose, 10 mM MES, 0.5% agarose L03 (TaKaRa Bio), 50 µg/mL ampicillin, 20 µg/mL thiabendazole in CYM medium, pH 6.5) at 42 °C, and poured 10 Petri dishes (9 cm diameter). The dishes were incubated at 30 °C for 2 days, 7.5 mL of regeneration medium containing 40 µg/mL hygromycin instead of thiabendazole was overlaid to each dish. Regenerated hyphal clones that appeared in 7 days incubation were picked and subcultured in 20 µg/mL hygromycin containing PDA medium. Genome PCR of regenerated clones were done with specific primers of *Tvfdh* ORF and *Tvgpd* terminator sequences (5’- gagctgctcaagagcttcaag -3’/5’-ggtttcgtttgtggcagagatg-3’), and PCR-positive clones were designated as F1-F38 strains. These transformed strains were employed to experiments, H_2_ production from cedar and effect of supplementation of organic acids to H_2_ production.

## 3 Results

### 3.1 H_2_ generation from wood by wood-rot fungi

**Figure 1:**
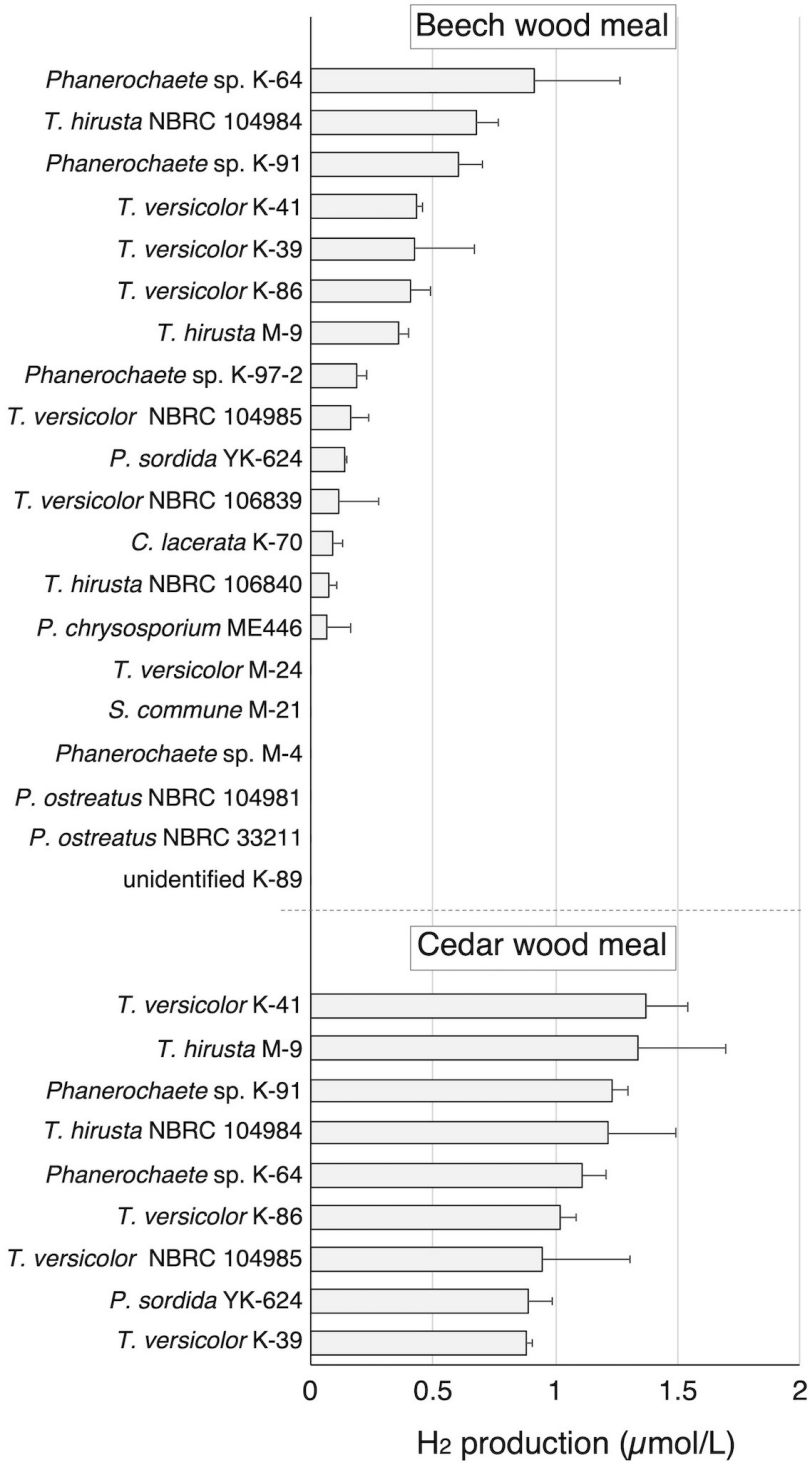
H_2_ production in beech and cedar wood meal cultures inoculated with white-rot fungi during 14 day incubation with sealing. Values are means ± standard deviation (n=3).

To investigate the H_2_-production activity of wood-rot fungi, 12 strains of wood-rot fungi (K and M strains) isolated from naturally decaying wood were inoculated on beech wood meal. During limited O_2_ supply incubation following aerobic cultivation, a small but clear H_2_ peak was observed on GC-TCD analysis of the headspace gas of several beech wood meal cultures (**Figure 1**). H_2_ was produced by 8 of 12 tested strains, and classification of those strains was attempted based on the ITS sequences. All 8 strains were found to belong to the Polyporales (1 *Ceriporia* strain, 3 *Phanerochaete* strains, and 4 *Trametes* strains). Therefore, 4 newly described *Trametes* fungi (2 strains each of *T. versicolor* and *T. hirsuta*) and 2 *Phanerochaete* fungi (*P. chrysosporium* and *P. sordida*) were tested in the same way. In addition, an Agaricales fungus (*P. ostreatus*) was also tested as a comparison. Although some strains of Polyporales fungi did not show H_2_ production, all strains showing H_2_ production belonged to the Polyporales. There were differences in the amount of H_2_ produced from beech wood meal among strains of same species.

Nine of the 14 H_2_-producing strains were then investigated for H_2_ production on cedar wood meal. All tested strains produced H_2_ from cedar in amounts greater than produced from beech. Because *T. versicolor* K-41 showed the highest H_2_ production level (1.36 µmol/L in headspace gas) in cedar wood meal culture (**Figure 1**), elucidation of H_2_ production mechanism of this fungus was attempted.

### 3.2 Characterization of H_2_ production activity of *T versicolor* K-41

**Figure 2:**
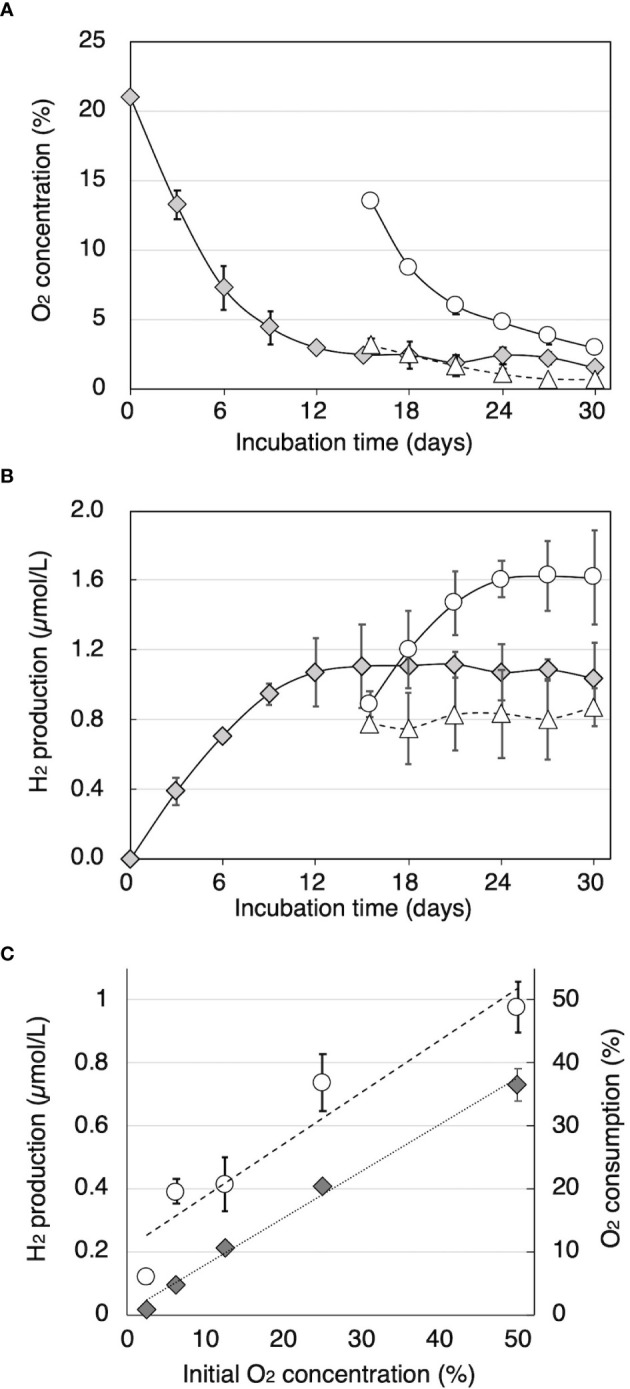
The relationship between O_2_ concentration and H_2_ production in sealed cedar wood meal cultures of *T. versicolor* K-41. Time courses of **(A)** O_2_ concentration and **(B)** H_2_ concentration in the headspace gas were monitored in sealed cultures (gray diamonds) and cultures flushed with 10 mL of O_2_ or N_2_ (black circles and white circles, respectively). **(C)** O_2_ consumption (black diamonds) and H_2_ production (white circles) were measured after 2-week incubation with O_2_ and N_2_ adjusted to the specified oxygen concentration. The values are the mean ± standard deviation (n=3).

Time courses of O_2_ consumption and H_2_ production by cultures of *T. versicolor* K-41 on cedar wood meal during cultivation after sealing were traced and shown in **Figure 2**. The O_2_ concentration in the headspace began to decrease immediately after sealing (**Figure 2A**). The concentration fell 2.9% by day 12, and thereafter, little O_2_ was consumed. By comparison, H_2_ production was observed at day 3, and production continued until 12 days of incubation. After termination of H_2_ production after 15 days, part of the headspace was replaced with pure O_2_ or N_2_ gas. Although no effect on H_2_ production was observed following replacement with N_2_, H_2_ production re-started following O_2_ replacement (**Figure 2B**). However, H_2_ production stopped again after the O_2_ was consumed. Effect initial O_2_ concentration on H_2_ production is shown in **Figure 2C**. The result indicated that higher initial O_2_ consumption and H_2_ production were well correlated with initial O_2_ concentration (R2 = 0.983 and 0.863, respectively).

**Figure 3:**
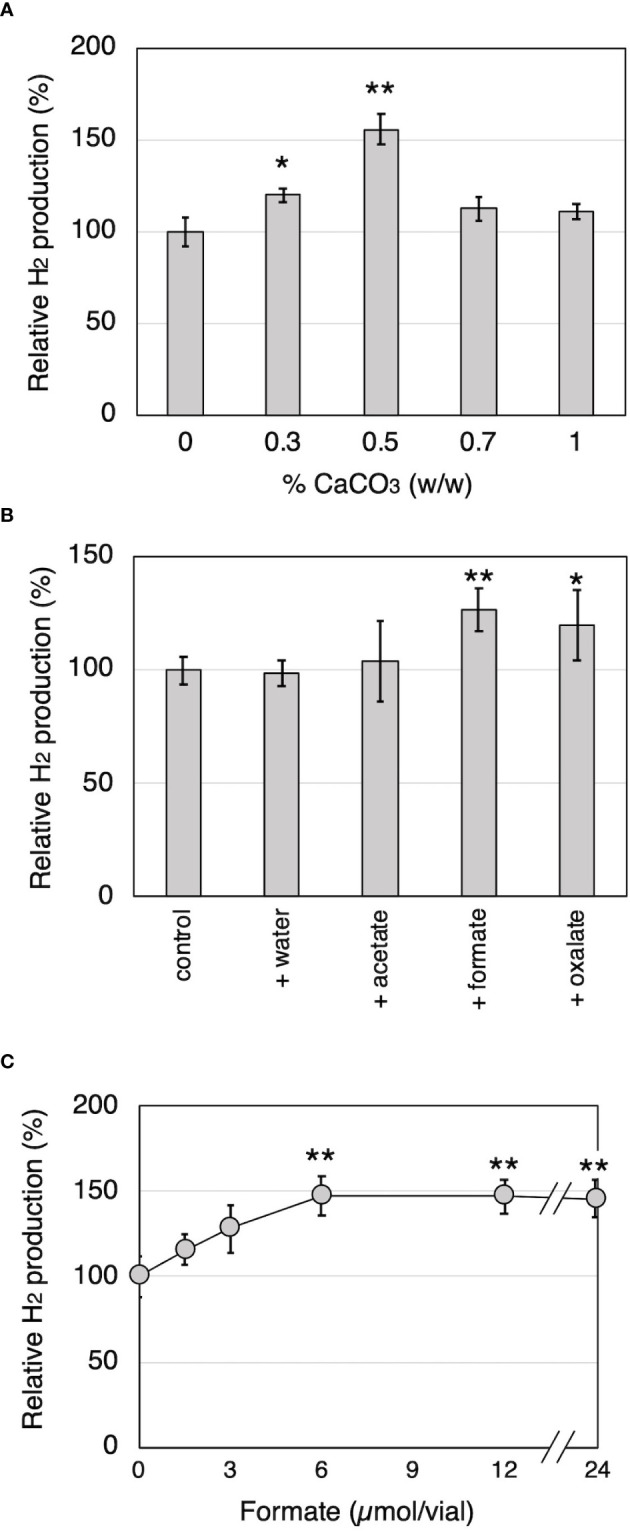
The effects of CaCO_3_ addition and organic acid supplementation on H_2_ production by *T. versicolor* K-41 in cedar wood meal cultures. The effects of **(A)** CaCO_3_ mixed ratio to wood medium, and **(B)** organic acid supplementation prior sealing. **(C)** The relationship between H_2_ production and amount of formate added to the wood culture. H_2_ production was measured on the 14th day after sealing. Asterisks indicate significant differences compared to the control (without any supplementation, * *P*<0.05, ** *P*<0.01). Values are mean ± standard deviation (n=3).

To investigate the relationship between H_2_ production and organic acid production during wood decay by *T. versicolor* K-41, H_2_ production from cedar wood meal containing 0-1.0% CaCO_3_ was investigated. It is well known that CaCO_3_ promotes the production of oxalate and other organic acids generated in the TCA/glyoxylate cycles of basidiomycetes (Takao, 1965). As shown in **Figure 3A**, *T. versicolor* K-41 showed significantly higher H_2_ evolution in cedar meal containing 0.3 and 0.5% CaCO_3_ than in culture without CaCO_3_; in particular, 1.5 times higher H_2_ production was observed in the culture containing 0.5% CaCO_3_. Therefore, to clarify involvement of organic acids, oxalate, formate, and acetate were added to 5-day-old cedar wood cultures of *T. versicolor* K-41 just before sealing, and H_2_ production was then measured. While no difference was observed in H_2_ production between the control and water- or acetate-supplemented cultures, oxalate and formate supplementation increased H_2_ production to 120% and 127% compared with the control (**Figure 3B**). H_2_ production increased depending on the level of formate supplementation, reaching a plateau at 6 µmol/flask (**Figure 3C**). In contrast, no H_2_ evolution was observed regardless of CaCO_3_ addition in T-medium, even though the oxalate and formate concentrations in the medium were increased by CaCO_3_ addition (**Table 1**). Thus, H_2_ production by *T. versicolor* K-41 may be a specific phenomenon during wood decay and may be related to the metabolism of organic acids, especially formate.

**Table 1:** Cultivation profiles of *T. versicolor* K-41 in liquid medium with or without CaCO_3_.

	control	0.1% CaCO_3_
mycelia dry weight (mg)	121.3 ± 2.6	122.6 ± 4.2
oxygen remaining (%)	2.1 ± 0.5	2.2 ± 0.8
organic acid in fluid (mmol/L)		
oxalate	4.4 ± 0.3	10.9 ± 1.6**
formate	0.5 ± 0.1	1.3 ± 0.1 **

The cultures after 14 days incubation with sealing (limiting O2 supply) following 5 days aerobic pre-cultivation were used for the analyses. Asterisks indicate significant differences to wild type or no supplementation control (** P<0.01). Values are means ± standard deviation (n=3).

### 3.3 Estimation of H_2_-producing pathway of *T. versicolor*

**Figure 4:**
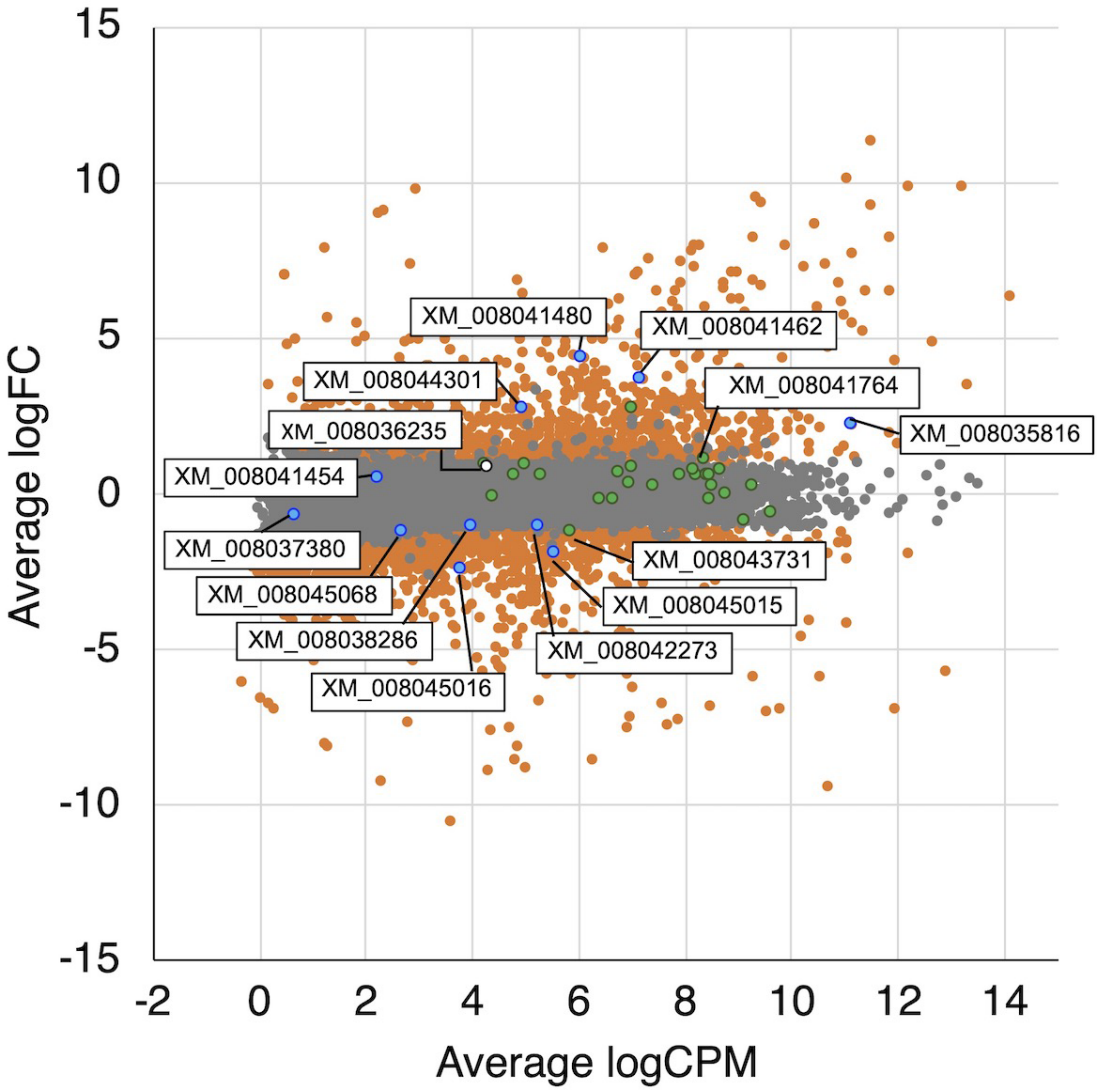
MA plot of the normalized data. Each dot represents a single gene, and significantly differentially expressed genes are colored by orange. The genes related TCA/glyoxylate cycle and oxalate metabolism are colored by green and blue, respectively. A white dot is indicated a hydrogenase-like gene.

To identify differences in gene expression between H_2_-producing and non–H_2_-producing conditions, a differential expression analysis between wood and liquid cultures was performed. These culture conditions were completely different, indicating that gene expression patterns are expected to differ significantly. Simultaneously, it was predicted a possibility of differences in the expression of H_2_ production-related genes due to clear differences in H_2_ production. Therefore, a differential gene expression analysis under these conditions was conducted. A large number of genes showed differential expression (logFC >1.0 or <−1.0, FDR <0.05); a total of 1,106 and 1,256 genes were up-regulated in wood medium and liquid medium, respectively (**Figure 4** and **Supplementary Tables 1**, **2**). In *T. versicolor* K-41 cultivated on wood medium, many genes encoding cellulolytic and ligninolytic enzymes were up-regulated compared with liquid medium (**Supplementary Table 1**). In contrast, several genes encoding hydrophobin and amylase-type enzymes were down-regulated on wood medium (**Supplementary Table 2**). GO enrichment analysis indicated that cellulose metabolic process, peroxidase, processes related to oxidative stress, cation uptake, and peptidase activities were over-represented in wood medium (**Supplementary Table 3**). These results suggest that *T. versicolor* K-41 initiates defensive mechanisms for ROS and radicals generated during the wood decay process.

**Figure 5:**
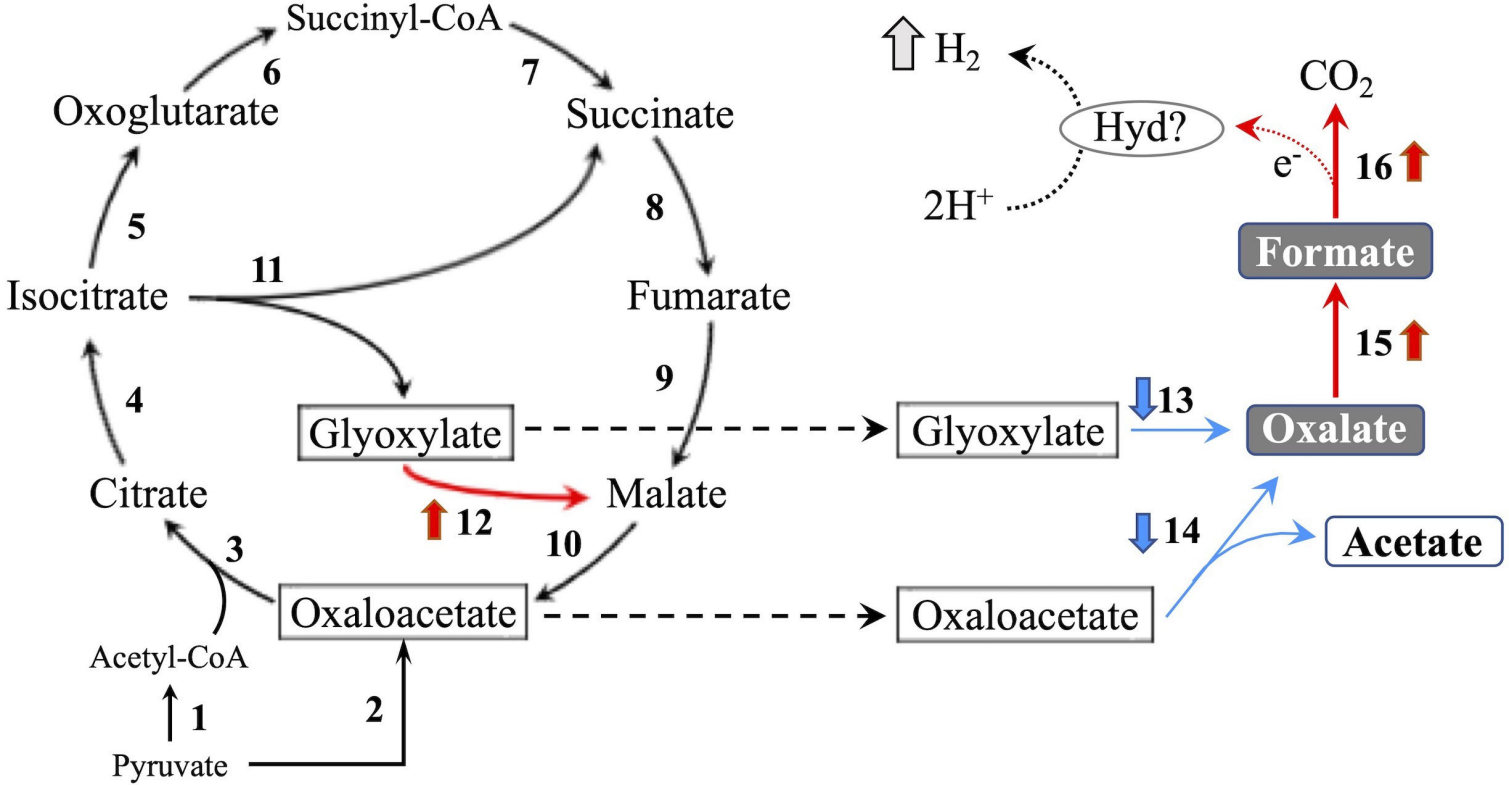
TCA/glyoxylate cycle and proposed H_2_ production pathway in *T. versicolor* K-41. Numbers beside arrows indicate enzymes catalyzing the reactions; 1: pyruvate dehydrogenase complex, 2: pyruvate carboxylase, 3: citrate synthase, 4: aconitate hydratase, 5: isocitrate dehydrogenase, 6: oxoglutarate dehydrogenase complex, 7: succinate-CoA ligase, 8: succinate dehydrogenase, 9: fumarate hydratase, 10: malate dehydrogenase, 11: isocitrate lyase, 12: malate synthase, 13: glyoxylate dehydrogenase and D/L-lactate dehydrogenase, 14: oxaloacetate acetylhydrolase, 15: oxalate decarboxylase (ODC), and 16: formate dehydrogenase (FDH). Bold upward and downward arrows indicate up- and down-regulated genes in wood culture compared with liquid culture, respectively. Protons may be reduced to H_2_ *via* catalytic reaction of an as yet unknown hydrogen-producing enzyme (Hyd)? using electrons produced during formate metabolism.

In the glyoxylate cycle, only the malate synthase gene (XM_008035809.1) was up-regulated on wood medium (**Figure 5** and **Table 2**). No induction of TCA cycle (GO:0006099) genes was observed compared with liquid culture, although dehydrogenase E1 and transketolase domain-containing protein 1 (XM_008043731.1) were down-regulated, and succinate dehydrogenase cytochrome b560 subunit (XM_008041764.1) was up-regulated in wood culture (**Figure 4**, **Table 2** and **Supplementary Table 3**). Malate synthase (XM_008035809.1) of the glyoxylate cycle was up-regulated on wood medium. As shown in **Figure 5** and **Table 2**, possible oxalate-producing enzymes were found in the annotations of the *T. versicolor* FP-101664 SS1 genome: a glyoxylate dehydrogenase (XM_008045016.1) and D- and L-lactate dehydrogenases (XM_008037380.1 and XM_008038286.1) that produce oxalate from glyoxylate (Davies and Asker, 1983; Munir et al., 2001). Although a gene encoding oxalaoacetate, which produces oxalate from oxaloacetate, was not found in the genome, XM_008045015 (annotated as a phosphoenolpyruvate/pyruvate domain-containing protein) showed significant similarity (identity 88.1%, query coverage 86.0%) to the oxaloacetate acetylhydrolase of *Fomitopsis palustris* (accession: AB690578.1, Hisamori et al., 2013). The expression of all of these putative oxalate-producing enzymes in wood culture was clearly lower than the expression in liquid culture. Five oxalate decarboxylase (ODC) genes have been annotated in the *T. versicolor* genome (**Table 2**). Two of these ODCs were up-regulated (XM_008041462.1 and XM_008041480.1) in wood culture, and two others were down-regulated (XM_008042273.1 and XM_008045068.1). Two highly expressed ODC genes were markedly up-regulated (**Figure 4**); therefore, all ODC genes appear to be up-regulated. Two formate dehydrogenase (FDH) genes (XM_008035816.1 and XM_008044301.1) showed expression levels ≥4 times higher in wood culture than in liquid culture. The transcript XM_008036235.1, annotated as an iron hydrogenase, showed relatively higher expression in wood culture compared with liquid culture (logFC=0.89, P=0.053, and FDR=0.125).

**Table 2:** Fold-change in expression of genes relating to the TCA/glyoxylate cycle and oxalate metabolism in wood culture compared to liquid culture.

Enzyme name^1^/gene ID	Annotation	logFC	logCPM	P value	FDR	GOterm	KOterm
1) Pyruvate dehydrogenase complex							
XM_008037889.1	mitochondrial pyruvate dehydrogenase E1 component beta subunit	0.59	8.23	0.064	0.145		K00161
XM_008034705.1	pyruvate dehydrogenase	0.84	8.14	0.013	0.043		K00627
2) Pyruvate carboxylase							
XM_008040683.1	pyruvate carboxylase	-0.80	9.11	0.016	0.050		K01958
3) Citrate synthase							
XM_008038329.1	citrate synthase-like protein	0.39	6.95	0.184	0.322		K01647
XM_008041170.1	citrate synthase	0.61	8.42	0.057	0.134	GO:0006099	K01647
XM_008040953.1	peroxysomal citrate synthase	0.61	4.78	0.039	0.100	GO:0006099	K01647
4) Aconitate hydratase							
XM_008034216.1	aconitate hydratase	0.95	4.98	0.001	0.006	GO:0006099	K17450
XM_008042398.1	aconitate hydratase	0.27	9.27	0.457	0.613	GO:0006099	K01681
5) Isocitrate dehydrogenase							
XM_008045165.1	isocitrate dehydrogenase	0.92	7.03	0.001	0.006		K00031
XM_008036665.1	hypothetical protein	-0.18	6.66	0.552	0.699	GO:0006099	
XM_008037535.1	hypothetical protein	-0.17	6.40	0.563	0.709	GO:0006099	
6) Oxoglutarate dehydrogenase complex							
XM_008039342.1	2-oxoglutarate dehydrogenase E1 component	-0.11	4.37	0.712	0.825	GO:0006099	K00164
XM_008043264.1	2-oxoglutarate dehydrogenase E1 component	0.06	8.75	0.847	0.918	GO:0006099	K00164
XM_008043731.1	dehydrogenase E1 and transketolase domain-containing protein 1	-1.14	5.87	0.000	0.000	GO:0006099	K15791
XM_008043611.1	dihydrolipoamide succinyltransferase	0.26	7.40	0.362	0.522	GO:0006099	K00658
7) Succinate-CoA ligase							
XM_008033951.1	succinate-CoA ligase	0.70	6.73	0.009	0.032		K01899
XM_008034755.1	succinate-CoA ligase	0.62	7.89	0.043	0.108	GO:0006099	K01900
8) Succinate dehydrogenase							
XM_008037945.1	succinate dehydrogenase	0.31	8.52	0.335	0.494	GO:0006099	K00234
XM_008041764.1	succinate dehydrogenase cytochrome b560 subunit	1.10	8.35	0.001	0.003	GO:0006099	K00236
XM_008043277.1	succinate dehydrogenase iron-sulfur subunit	0.63	8.47	0.051	0.122	GO:0006099	K00235
9) Fumarate hydratase							
XM_008034924.1	fumarate hydratase	-0.17	8.49	0.620	0.754	GO:0006099	K01679
10) Malate dehydrogenase							
XM_008037733.1	malate dehydrogenase	0.82	8.68	0.023	0.068	GO:0006099	K00026
XM_008038723.1	malate dehydrogenase	-0.62	9.61	0.056	0.131	GO:0006099	K00026
11) Isocitrate lyase							
XM_008039384.1	isocitrate lyase	0.58	5.28	0.211	0.356		K01637
XM_008034471.1	isocitrate lyase	0.97	4.23	0.001	0.007		K01637
12) Malate synthase							
XM_008035809.1	malate synthase	2.81	7.03	0.000	0.000	GO:0006097	K01638
13) Oxalate producing enzymes							
XM_008045016.1	glyoxylate dehydrogenase	-2.37	3.80	0.000	0.000		K00101
XM_008037380.1	L-lactate dehydrogenase	-0.71	0.69	0.250	0.402		
XM_008038286.1	D-lactate dehydrogenase cytochrome oxidoreductase	-1.01	3.97	0.000	0.002		K00102
14) Oxaloacetate acetylhydrolase							
XM_008045015.1	Phosphoenolpyruvate/pyruvate domain-containing protein	-1.88	5.54	0.001	0.004		
15) Oxalate decarboxylase (ODC)							
XM_008041454.1	oxalate decarboxylase	0.57	2.26	0.156	0.285	GO:0033609	
XM_008041462.1	oxalate decarboxylase	3.74	7.17	0.000	0.000	GO:0033609	
XM_008041480.1	oxalate decarboxylase	4.45	6.08	0.000	0.000	GO:0033609	
XM_008042273.1	Bicupin oxalate decarboxylase/oxidase	-1.06	5.22	0.001	0.003	GO:0033609	K01569
XM_008045068.1	oxalate decarboxylase	-1.23	2.68	0.001	0.006	GO:0033609	
16) Formate dehydrogenase (FDH)							
XM_008035816.1	NAD-dependent formate dehydrogenase	2.29	11.13	0.000	0.000	GO:0008863	K00122
XM_008044301.1	NAD-dependent formate dehydrogenase	2.79	4.95	0.000	0.001	GO:0008863	K00122
Hyd)? Hydrogenase-like gene							
XM_008036235.1	iron hydrogenase	0.89	4.28	0.053	0.125		

^1^ Numerical values in front of gene names are corresponded to **Figure 3**.

**Figure 6:**
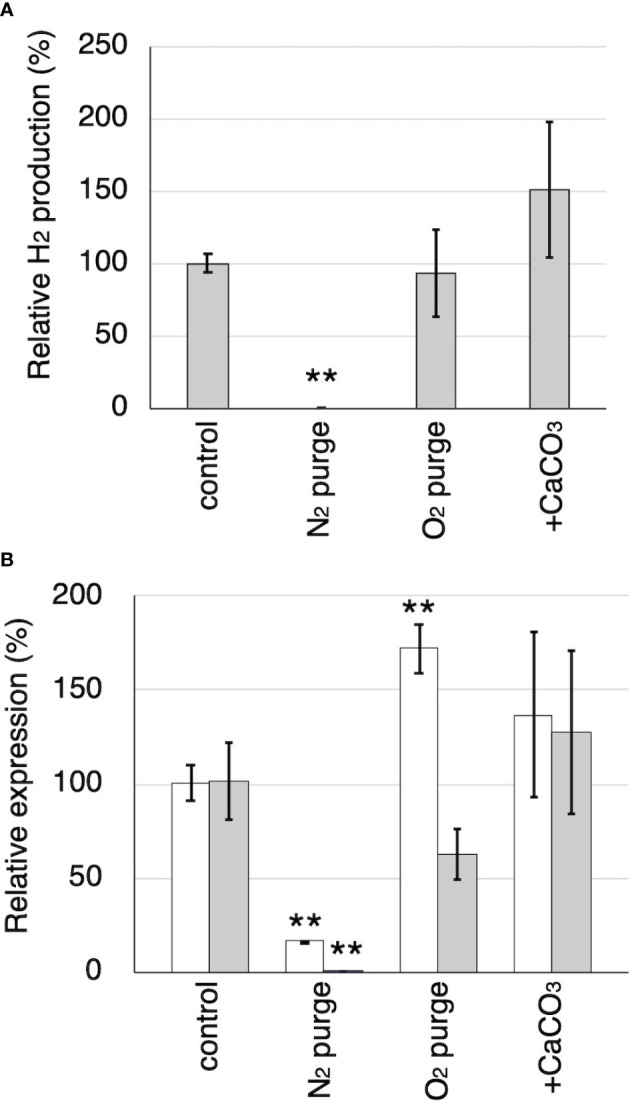
The effects of N_2_ and O_2_ purging, CaCO_3_ addition to the cultures on H_2_ production and the relative expression of *Tvhyd* and *Tvfdh*. **(A)** Relative H_2_ production in 7th day compared to the control. **(B)** Relative expression of *Tvhyd* (white) and *Tvfdh* (gray) determined using ΔΔCq method with *Tvgpd* as a reference gene. Asterisks indicate significant differences compared to the control (** *P*<0.01). Values are mean ± standard deviation (n=3).

*Tvfdh* (encoding a formate dehydrogenase) and *Tvhyd* (annotated as an iron hydrogenase) were identified from the genome and cDNA of *T. versicolor* K-41. Relative production of H_2_ and relative expression of *Tvhyd* and *Tvfdh* at 7 days after sealing in N_2_- and O_2_-purged wood cultures and wood culture containing 0.5% CaCO_3_ are shown in **Figures 6A, B**. O_2_ purge did not affect H_2_ production; however, H_2_ was undetectable in N_2_-purged cultures (**Figure 6A**). No difference was observed in H_2_ production between the control and O_2_-purged samples. This is likely due to the presence of residual O_2_ in the samples during the early stages after sealing, resulting in H_2_ production still proceeding in both samples. CaCO_3_ addition increased H_2_ production to approximately 150%, as shown in **Figure 3A**. Relative *Tvhyd* expression was significantly lower (17%) in N_2_-purged cultures and higher (172%) in O_2_-purged cultures compared with the control. In the case of *Tvfdh*, this gene showed significantly lower expression (less than 1%) in N_2_-purged cultures and tended to exhibit lower expression (62%, P=0.051) in O_2_-purged cultures. Although there were no significant differences in the expression levels of *Tvhyd* and *Tvfdh* on CaCO_3_-supplemented cedar culture compared with the control, the expression levels of both genes appeared to be higher. There was a correlation between the *Tvfdh* expression level and H_2_ production (*r*=0.645); therefore, the relationship between *Tvfdh* and H_2_ production by *T. versicolor* K-41 was evaluated.

### 3.4 Effect of self-recombination of *Tvfdh* on H_2_ production

**Figure 7:**
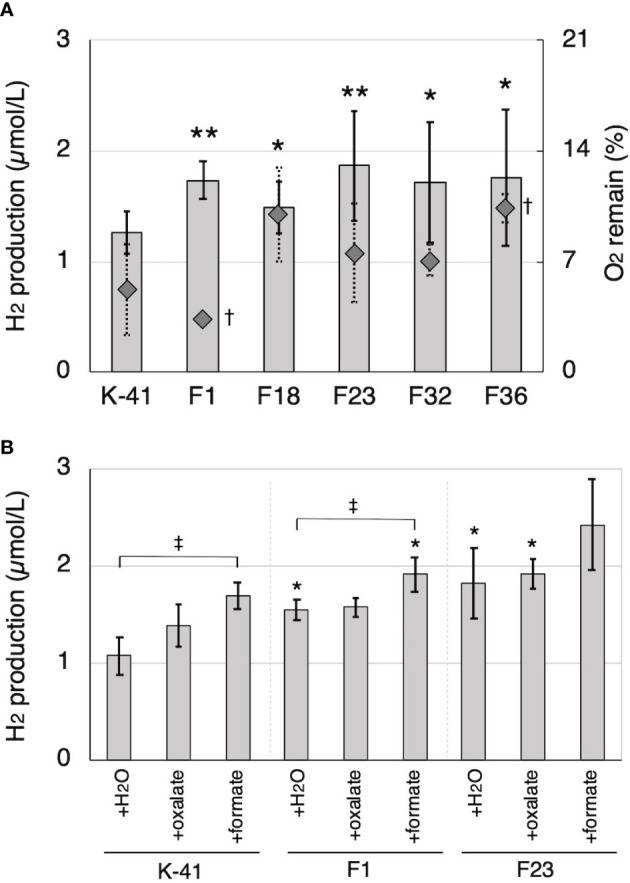
H_2_ production by *Tvfdh* self-recombination transformants. **(A)** H_2_ production and remaining O_2_ in cedar wood meal cultures of wild-type strain (K-41) and transformants (F strains). **(B)** The effects of oxalate and formate supplementation to H_2_ production of K-41 and transformants. O_2_ and H_2_ productions were measured on the 14th day after sealing. Asterisks (* and **) indicate significant differences (P<0.05 and 0.01) in H_2_ production compared to the wild type (plus same supplementation), respectively. Dagger (†) indicates significant difference in O_2_ remaining compared to the wild type (P<0.05). Double dagger (‡) indicates significant difference (P<0.05) compared to the H_2_O supplementation control of the same strain. Values are mean ± standard deviation (n=3).

Cell-free extract obtained from IPTG-induced *E. coli* retaining *Tvfdh* cDNA in pCold I showed clear NADH formation dependent on formate dehydrogenase activity (data not shown). Thus, construction of *Tvfdh* self-recombinant transformants of *T. versicolor* K-41 was attempted. A total of 38 self-recombinant strains were recovered by co-transformation, and all of these strains were employed to evaluate O_2_ consumption during 2 weeks of incubation on cedar wood meal culture to estimate growth on wood meal. Five transformants showing 50% or higher O_2_ consumption were selected. The remaining 33 strains showed approximately 30% or less O_2_ consumption, indicating that these strains probably grow slowly on cedar wood medium. The amount of H_2_ production and remaining O_2_ concentration after 14 days of incubation following sealing of cedar wood cultures inoculated with 5 selected strains were shown in **Figure 7A**. All selected strains showed a higher amount of H_2_ in the headspace. Furthermore, the effect of oxalate and formate supplementation on H_2_ production of *Tvfdh* over-expressing strains was investigated (**Figure 7B**). The wild-type strain exhibited improved H_2_ production by the addition of oxalate and formate, and formate supplementation in particular showed a clear effect, as shown in **Figure 3**. Transformants showed higher H_2_ production in cultures with either supplementation than did the wild-type strain with the same supplementation. In contrast, formate supplementation (3 µmol/vial) improved H_2_ production by the transformants, whereas oxalate supplementation (3 µmol/vial) did not have a clear effect on H_2_ production (**Figure 7B**).

## 4 Discussion

Hydrogen and methane gases hold promise as next-generation fuels. Biologically, both gases are produced by anaerobic microorganisms. Recent research revealed that eukaryotes, including animals, plants, and fungi, produce methane during responses to stressors such as ROS, even in the presence of O_2_ (Liu et al., 2015). In the case of biohydrogen, many reports have described H_2_ production from not only bacteria but also anaerobic eukaryotes. Hydrogenase-like genes are widely distributed among eukaryotes, including higher eukaryotes; however, the functions of these genes are still unknown (Horner et al., 2002). Some reports have described H_2_ production by higher plants (e.g., Torres et al., 1986; Jin et al., 2013). Although the physiological roles of H_2_ in higher eukaryotes remain unclear, it is thought that H_2_ acts as an antioxidant and signaling molecule in higher plants and animals and improves tolerance to ROS (e.g., Itoh et al., 2011; Li et al., 2018). White-rot fungi produce a variety of radicals and ROS during the wood decay process (ten Have and Teunissen, 2001), and they may also have antioxidative self-defense mechanisms.

Based on these observations, it is hypothesized that white-rot fungi produce H_2_ as an antioxidant that protects against oxidative stressors such as ROS and radicals that are generated during the wood decay process. In tightly sealed wood cultures, a peak of H_2_ on GC analysis was observed in the headspace gas of samples of more than half of the white-rot fungi species tested (**Figure 1**). While this was a very novel and interesting finding, the respective amounts and efficiencies of H_2_ production were less than 1/1000 of bacterial H_2_ production from lignocellulose (Ren et al., 2016). Therefore, H_2_ production by white-rot fungi during wood decay is likely to be a secondary metabolic reaction rather than primary metabolism associated with energy production. It is possible that a portion of the produced H_2_ is consumed to reduce the toxicity of radicals and ROS generated during wood decay under aerobic conditions. The H_2_ production properties of *T. versicolor* K-41, which showed the highest H_2_ production on wood medium, were thus investigated further. After sealing of the culture vials, this fungus consumed O_2_ in the headspace *via* respiration, and H_2_ production was only observed during O_2_ consumption (**Figures 2A, B**). If O_2_ was re-supplied to the headspace of culture vials after O_2_ consumption ceased, the fungus resumed H_2_ production. Additionally, H_2_ production was well correlated with O_2_ concentration (**Figure 2C**). Thus, these results indicated that white-rot fungi emit H_2_ during aerobic respiration but not anaerobic conditions, a property that contrasts markedly with that of bacterial H_2_ production. These results also suggested that there is a relationship between H_2_ production and wood decay by white-rot fungi.

Some H_2_-producing bacteria are capable of utilizing short-chain organic acids such as formate, acetate, and lactate for H_2_ production (Barbosa et al., 2001; Matsumoto and Nishimura, 2007; McDowall et al., 2014). It has also been shown that hydrogenosomes, organelles found in a wide variety of anaerobic eukaryotes, produce H_2_ during pyruvate or malate metabolism (Davidson et al., 2002). These data suggest that organic acids have a significant effect on microbial hydrogen production. In environments with a high C/N ratio, such as wood, it is thought that white-rot fungi dispose of excess carbon as oxalate or other metabolites. Most oxalate is probably produced intracellularly from intermediates (oxaloacetate and glyoxylate) of the TCA and glyoxylic acid cycles (Mäkelä et al., 2002). Although oxalate accumulation in wood culture correlates well with fungal growth and ligninolytic manganese peroxidase activity in some white-rot fungi, including *T. versicolor*, white-rot fungi readily decompose excessive oxalate *via* intra/extracellular metabolism in order to avoid its toxic effect ((Dutton et al., 1993; Mäkelä et al., 2002). Oxalate is degraded to CO_2_ *via* formate by intracellular ODC and FDH and also degraded to CO_2_ by extracellular peroxidase systems (Shimada et al., 1994). These pathways may enable white-rot fungi to control the concentration of intra/extracellular oxalate to maintain physiological conditions. The concentration of oxalate in the medium was shown to increase after addition of CaCO_3_ to white-rot fungi cultures (Takao, 1965). The data presented here provide novel insights into the relationship between oxalate metabolism and H_2_ production, because higher H_2_ production was observed in cedar wood cultures supplemented with CaCO_3_, oxalate, and formate compared with control cultures (**Figures 3**, **7**). These results suggested that metabolism of organic acids, especially formate, is involved in H_2_ production by *T. versicolor* K-41.

No hydrogen production was observed in the liquid culture, even though the addition of CaCO_3_ enhanced extracellular oxalate accumulation (**Table 1**). RNA-seq analyses showed that *T. versicolor* K-41 exhibited lower oxalate production and higher oxalate metabolic activity in wood culture compared with liquid culture. As shown in **Table 2**, *T. versicolor* K-41 promoted the expression of oxalate metabolic enzymes, ODCs and FDHs, in wood culture. In contrast, oxalate-producing enzymes were suppressed. In addition, *T. versicolo*r K-41 appeared to avoid accumulation of toxic organic acids, glyoxylate, oxalate, and formate, as only malate synthase was upregulated among the enzymes in the glyoxylate cycle. These result support the hypothesis that oxalate/formate metabolism is involved in H_2_ production in wood culture. However, this data did not clarify the relationship between the expression of hydrogenase-like genes and H_2_ production by *T. versicolor* K-41. Thus, these experiments indicate that molecular hydrogen synthesis is not the rate-limiting step in the H_2_ production system in this fungus.

Accordingly, it was investigated the relationship between the expression of a hydrogenase-like gene (*Tvhyd*) and FDH gene (*Tvfdh*) and H_2_ production under different O_2_ conditions or CaCO_3_ supplementation (**Figure 6**). N_2_ purge showed clear effects, as low O_2_ conditions completely suppressed H_2_ production and inhibited *Tvfdh* expression. This result suggests that *Tvfdh* expression is likely more correlated with H_2_ production than *Tvhyd*. Therefore, formate metabolism catalyzed by TvFDH is probably involved in H_2_ production. *Tvfdh* self-recombination strains that maintained O_2_ consumption produced higher amounts of H_2_ in the headspace than the wild-type strain; however, transformants only exhibited improved H_2_ production following formate supplementation, unlike the wild type (**Figure 7**). These results suggest that the amounts of oxalate and formate or the associated metabolic activities are the rate-limiting step in H_2_ production by *T. versicolor* K-41. Therefore, a possible H_2_ production pathway for *T. versicolor* K-41 is proposed, as shown in **Figure 5**. Oxalate originating in the TCA/glyoxylate cycle is metabolized to form formate by intracellular ODC, and FDH oxidized formate to generate CO_2_, H^+^, and two electrons. from formate. Then, TvHYD or an as yet unknown hydrogenase perhaps produces H_2_ from protons by utilizing electrons produced in formate metabolism.

In this study, it was discovered that some white-rot fungi belonging to the Polyporales are capable of producing H_2_ during wood decay. In the case of *T. versicolor* K-41, which showed the highest H_2_ production, the fungus produced H_2_ under aerobic conditions, and oxalate/formate metabolism is likely linked to the H_2_ production system. In addition to these results, self-recombination of *Tvfdh* clearly improved H_2_ production in cedar wood culture, thus suggesting that TvFDH is involved in H_2_ production by this fungus. This novel finding of aerobic H_2_ production by an aerobic white-rot fungus opens new areas of inquiry in biohydrogen research. It is expected that be economically advantageous over anaerobic fermentation for process control if an aerobic H_2_ production process could be established. However, the current H_2_ production by white rot fungi is far below the commercially viable level. Additionally, there are many open questions remaining in terms of the mechanism of aerobic H_2_ production by white-rot fungi, the involvement of bacterial symbionts in H_2_ production by white-rot fungi has not been excluded, and the H_2_ production mechanism remains unclear. In future studies, identification of the enzyme producing H_2_ and the underlying mechanism will be attempted in order to shed light on the physiological function of H_2_ production in white-rot fungi.

## Data availability statement

The datasets presented in this study can be found in the DDBJ repository, accession number DRA014108 (https://ddbj.nig.ac.jp/resource/sra-submission/DRA014108).

## Author contributions

TM screened H_2_-producing fungi, analyzed gene expression, interpreted experiments, and wrote the manuscript. ST analyzed the effect of oxygen and self-recombinant strains. AS performed characterization of H_2_ production activity. MA determined gene and cDNA sequences and contributed screening. RK and YY contributed to the construction of recombinant strains. HD performed RNA-seq and analyzed the data. HK proposed the H_2_ production pathway. HH designed and interpreted the experiments and wrote the manuscript. All authors contributed to the article and approved the submitted version.
